# Cardiovascular magnetic resonance 4D flow derived aortic and pulmonary wall shear stress in pediatric patients with repaired tetralogy of Fallot

**DOI:** 10.3389/fped.2025.1623218

**Published:** 2025-10-30

**Authors:** A. Nyström, F. Dangardt, M. Synnergren, H. Wåhlander, J. Sunnegårdh, P.-A. Svensson, K. Lagerstrand, C. De Lange

**Affiliations:** ^1^ Department of Pediatric Radiology, Queen Silvia Childrens’ Hospital, Sahlgrenska University Hospital, Gothenburg, Västra Götaland, Sweden; ^2^ Institute of Clinical Sciences, Sahlgrenska Academy, University of Gothenburg, Gothenburg, Västra Götaland, Sweden; ^3^ Children’s Heart Center, Sahlgrenska University Hospital, Gothenburg, Västra Götaland, Sweden; ^4^ Institute of Internal Medicine, Sahlgrenska Academy, University of Gothenburg, Gothenburg, Västra Götaland, Sweden; ^5^ Department of Biomedical Engineering and Medical Physics, Sahlgrenska University Hospital, Gothenburg, Västra Götaland, Sweden

**Keywords:** ascending aorta, magnetic resonance imaging, tetralogy of Fallot, wall shear stress, pulmonary artery

## Abstract

**Background:**

Patients with repaired tetralogy of Fallot (rTOF) risk dilatation of the pulmonary artery (PA) and the ascending aorta (Ao). Ao and PA wall shear stress (WSS) assessed by cardiovascular magnetic resonance (CMR) 4D flow has been studied in adults while the relationships in children are less explored. The purpose of our study was to evaluate the association between WSS in Ao/PA and vascular remodelling in children with rTOF.

**Methods:**

Retrospective evaluation was performed in children with rTOF, with CMR including 4D flow, from January 2019 through 2022. Standardised measurements of WSS were performed in the PA and Ao in patients, and in pediatric controls. WSS values were compared and correlated to valvular patency, volumetrics, function, and demographics.

**Results:**

In the rTOF group (*n* = 43), median age of 14 years (range 0–18), most patients had a moderate to severe PA regurgitation (>10%, *n* = 40 and >25%, *n* = 31). Indexed mean Ao and PA diameters in the rTOF group were increased vs. controls (*p* < 0.001 and *p* = 0.009). Compared to controls (*n* = 15), the mean/peak PA WSS were significantly increased in rTOF (*p* < 0.001) but not significantly correlated to Ao/PA dilatation, PA regurgitation or to right ventricular function (R = −0.02–0.24, *p* = 0.5–0.9).

**Conclusion:**

Pediatric patients with rTOF revealed increased Ao/PA diameters and increased PA WSS compared to controls. There was a positive trend, but no association of WSS to Ao/PA dilatation nor PA regurgitation. Vascular remodelling is undoubtedly multifactorial in patients with rTOF. However, increased WSS could be a contributing factor to late complications in rTOF.

## Introduction

Children born with congenital heart disease (CHD) have seen improved survival rates during the last decades, owing to the introduction of modern surgical techniques, improved perioperative care, echocardiography and imaging (1). Patients born with Tetralogy of Fallot (TOF) have seen a drastic increase in long-term outcome; before surgery was available, these patients rarely reached adult age and had a poor quality of life (2–4).

One of the most common approaches for surgical correction of TOF is the transannular patch (TAP) technique. The pulmonary insufficiency that in most cases ensues TAP operation eventually leads to an enlarging right ventricle (RV), needing surgery to restore pulmonary valve patency and, hopefully, reverse or inhibit deterioration of ventricular function (5). Patients with repaired TOF (rTOF) face risks primarily from the pulmonary side of the circulation. Eventually, there is an increased risk of reduced left ventricular (LV) function, ascending aorta dilatation and aneurysm formation (6, 7).

Present guidelines regarding timing for pulmonary valve replacement (PVR) concern RV volume and function, pulmonary artery (PA) regurgitant fraction and the impact of the LV (8, 9). Advanced cardiovascular magnetic resonance imaging (CMR) four dimensional (4D) flow derived parameters are still under development and further validation is needed before they can be a fully established part of the clinical evaluation of rTOF.

CMR has emerged as a robust, reproducible and accessible method for diagnosis and follow-up in CHD (10, 11). CMR can provide information on morphology, cardiac function, blood flow and myocardial tissue characterization. Traditionally, flow measurements have been performed with CMR 2D-phase contrast (2D-PC) methods, based on bipolar gradients causing phase shifts in moving spins that are proportional to their velocities. CMR 4D flow, using the same 2D-PC technique but sampled in a volume in three dimensions, has proved useful, especially among CHD patients owing to their more complex anatomy (12, 13). 4D flow CMR allows for retrospective analysis of a large amount of flow parameters, not only velocity and regurgitant fraction, but also energy loss, vorticity, eccentricity, and wall shear stress (WSS). Wall shear stress is defined as the frictional force that the flowing blood exerts on the vessel wall and has been shown to influence vascular dilatation and aneurysmal widening. Little is known about WSS in the pathogenesis of long-term complications of CHD, such as PA and ascending aorta (Ao) dilatation, and biventricular dysfunction resulting from TOF or resultant aortic disease (14, 15).

The goals of our study were to review 4D flow CMR in pediatric patients with rTOF and to evaluate the relationship between WSS and Ao/PA valvular disease, in a cohort corrected with TAP, where a high proportion of the patients had a pulmonary regurgitation. We also wanted to compare our results in the rTOF-group with a pediatric control group. The primary aim was to investigate if WSS in the PA and Ao of patients with rTOF are elevated already in childhood or adolescence and correlated with risk markers as increased ventricular volumes, decreased systolic function and increased vessel diameters. A secondary aim was to evaluate WSS and cardiac function in rTOF patients without clinical indications of complications. Can increased WSS indicate advanced complications in rTOF and thereby timepoint for treatment and reintervention?

## Methods

Ethical approval was obtained by the Swedish ethical review authority (ethical approval DnR 452-17 and DnR 2022-02704-01). Informed consent from caregivers and patients was waived.

### Study population

Children between 0 and 18 years of age were referred for a clinically indicated CMR examination to assess RV function, PA regurgitation and pulmonary perfusion, from January 2019 through 2022, and were retrospectively included. The control subjects consisted of adolescents referred for a clinically indicated CMR for suspected arrhythmogenic right ventricular cardiomyopathy, with a normal clinical work up and CMR without pathological findings. Patient demographics were registered from medical records.

### Cardiovascular magnetic resonance protocol

The examinations were performed on a 3T or 1.5T MRI scanner (3T Signa Architect or 1.5T Signa Artist, GE Medical systems, Waukesha, WI, USA) with a standardized clinical protocol in free breathing, including 4D flow (described in detail in Supplementary Material S1). During 2019–2020, all examinations (*n* = 19) were performed on 3T, due to insufficient quality of the 4D flow on the 1.5T scanner. During 2021–2022, *n* = 16 patients were examined on the 3T and *n* = 8 on the 1.5T scanner after software adaptations. Balanced steady-state free precession (bSSFP) sequences performed in free breathing were used for analysis of ventricular function. An ECG-gated, respiratory compensated and motion-corrected 4D PC CMR flow protocol was used for flow analysis. Velocity encoding (VENC) was optimized to avoid velocity aliasing and was chosen individually for each patient, based upon the highest velocity measured on echocardiography the same day or the day before the MRI examination, typically ranging from 125 to 350 cm/s. General anaesthesia without intubation or sedation was used when needed. The scan protocol lasted∼45 min, and the 4D flow sequence duration was 6–8 min.

### CMR analysis

**Figure 1:**
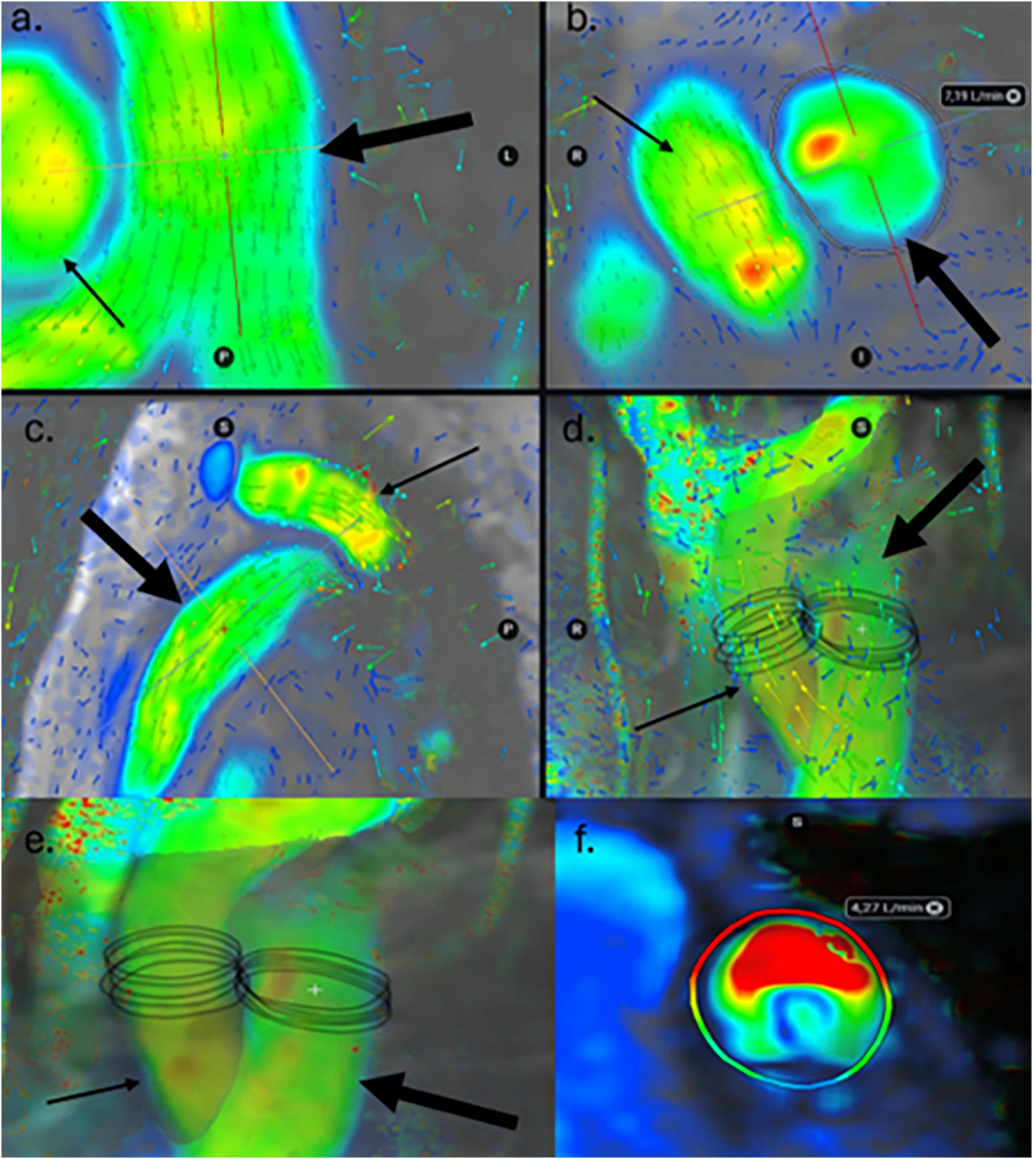
CMR 4D flow colour map in a control subject in multiplanar reconstruction at the level of the mid pulmonary artery (PA). The vector arrows in the vessels indicate the dominant flow direction and thus where the correct flow measurements should be placed and at what angle. Low velocities are shown as blue or green, and high as yellow or red. Thick arrow represents the pulmonary artery and thin arrow the ascending aorta. **(a)** transverse view, **(b)** coronal view, **(c)** sagittal view, and **(d)** volume rendering including vector arrows. **(e)** shows a close-up of a coronal view of volume rendering. The five closely spaced measurements of circumferential wall shear stress are shown in the ascending aorta and the pulmonary artery in one of the control subjects. **(f)** Circumferential wall shear stress for each measured level is displayed as a heatmap where high values are coloured red and low are blue, in this example from one of the patients with rTOF.

Examinations were reviewed and postprocessing was done offline by one pediatric radiologist (A.N) using the software Cardio AI (Tempus Medical/Arterys, Redwood Shores, CA, USA), where ventricular function and flow data were analysed. Ventricular function was analysed by semiautomatic and manually adjusted segmentation on bSSFP cine images in the short axis plane, in accordance with present guidelines (16). Flow was reconstructed from the CMR 4D flow volume using double-oblique planes perpendicular to the dominant flow direction, in the ascending aorta and in the main pulmonary artery (Figures 1a–d). For mitigating the influence of measurement noise 5 closely spaced (distance: 1 mm) manually traced measurements were performed; in the mid pulmonary artery and in the ascending aorta just above the level of the sinotubular junction (Figure 1e). The WSS value is an average of WSS in all voxels around the circumference of the vessel. The lumen contours were manually adjusted at all time frames of the cardiac cycle to provide best fitting to the vessel contour. Both peak and mean WSS were registered. Peak WSS is the highest WSS value measured in that vessel and can be in either systole or diastole. Mean WSS, also referred to as time-averaged WSS (TAWSS) is an average of all WSS values across all 20 or 30 time frames of the cardiac cycle. Vessel diameter derived from CMR 4D flow images were indexed to BSA, using the DuBois formula (17). Peak vessel diameter was measured the same way as WSS, using the highest diameter measured from all time frames; typically the highest values are found in systole. Mean vessel diameter is an average of the diameters of all time frames of the cardiac cycle. Wall shear stress was calculated by the software using velocity data from the 4D flow and a presumed blood viscosity of 3.5 mPas (Figure 1f).

### Statistical analysis

Continuous variables are presented as means ± standard deviation (SD) if normally distributed and otherwise as medians and ranges. Categorical variables are expressed as counts and percentages of the total. The data was tested for normal distribution, using the Kolmogorov—Smirnov and Shapiro–Wilk test. Categorical and continuous data were analysed as appropriate. The presented values of mean WSS and peak WSS were averaged over the five ROIs in both the Ao and PA and compared between groups using independent samples t-test. The correlations between parameters were assessed using Pearsons’ rank correlation test for continuous variables or Fisher's exact test used for categorical data and small samples. A *p*-value less than 0.05 was considered significant. All statistical analyses were performed in SPSS Statistics for Macintosh (IBM Corp. Released 2021. IBM SPSS Statistics for Macintosh, Version 28.0. Armonk, NY: IBM Corp)

## Results

Patient demographics are presented in Table 1. Forty-three patients with rTOF and 15 control subjects performed CMR. Fifteen of the rTOF patients and one of the control subjects were scanned under general anaesthesia. The majority of rTOF patients (*n* = 35) had undergone repair with TAP. The remaining 8 patients were operated with valve sparing techniques (*n* = 6) or had a PA homograft (*n* = 2). Out of the 43 rTOF patients, 29 had had their first operation done before the age of one year, nine after one year and five patients had an unknown operation date; in most cases due to operation in another country before adoption. Six patients had secondary pulmonary valve surgery or intervention where four patients were >13 years of age at the time of CMR. Median age at first procedure was 5 months (range 0–60). The patients in the rTOF group (*n* = 43) were median 12 years (range 0–18), and the controls (*n* = 15) were somewhat older, median 16 years (range 11–18) (*p* < 0.001) at time of CMR.

**Table 1:** Patient demographics and CMR data.

Demographics	Repaired tetralogy of fallot *n* = 43	Controls *n* = 15	*p*-value controls vs rTOF	95% confidence interval	Effect size cohen's d
Age (years)	11.6 ± 4.0	15.3 ± 1.7	<0.001	0.460 to 1.723	1.096
Gender (male/female %)	58/42%	67/33%	0.56	na	na
Body Surface Area (m/m^2^)	1.32 ± 0.4	1.7 ± 0.2	<0.001	0.508 to 1.777	1.147
CMR data (derived from 4D flow)					
Aorta peak diameter/Body Surface Area (mm/m^2^)	22.4 ± 6.2	16.5 ± 1.8	<0.001	−1.650 to −0.396	−1.028
Aorta mean diameter/Body Surface Area (mm/m^2^)	21.4 ± 6.1	15.5 ± 1.6	<0.001	−1.700 to −0.440	−1.074
Aorta vmax (m/s)	0.92 ± 0.29	1.26 ± 0.3	<0.001	0.490 to 1.758	1.129
Aortic regurgitation (%)	3 ± 7	1 ± 1	0.29	−0.907 to 0.290	−0.310
Aortic peak WSS (cPa)	122 ± 50	102 ± 29	0.14	−0.954 to 0.245	−0.357
Aortic mean WSS (cPa)	22 ± 7	19 ± 5	0.76	−1.118 to 0.089	−0.517
Pulmonary artery peak diameter/ Body Surface Area (mm/m^2^)	21.9 ± 7.3	18.6 ± 1.7	0.09	−1.290 to −0.071	−0.684
Pulmonary artery mean diameter/ Body Surface Area (mm/m^2^)	19.6 ± 6.6	16.6 ± 1.6	0.01	−1.273 to −0.55	−0.667
Pulmonary artery vmax m/s	1.13 ± 0.62	0.97 ± 0.27	<0.001	−2.019 to −0.716	−1.373
Pulmonary regurgitation %	33 ± 14	1 ± 1	<0.001	−3.475 to −1.898	−2.694
Pulmonary WSS peak (cPa)	180 ± 73	93 ± 29	<0.001	−2.012 to −0.711	−1.367
Pulmonary WSS mean (cPa)	41 ± 14	17 ± 4	<0.001	−2.656 to −1.248	−1.959
CMR data (derived from cine balanced steady state free precession (SSFP)					
LVEDVI (ml/m^2^)	73 ± 20	88 ± 15	0.003	0.224 to 1.458	0.845
LVESVI (ml/m^2^)	31 ± 10	37 ± 11	0.10	−0.068 to 1.141	0.539
LVEF (%)	57 ± 6.5	59 ± 5.8	0.20	−0.247 to 0.952	0.845
RVEDVI (ml/m^2^)	121 ± 23	87 ± 12	<0.001	−2.258 to −0.919	−1.595
RVESVI (ml/m^2^)	64 ± 16	41 ± 10	<0.001	−2.267 to −0.927	−1.603
RVEF (%)	47 ± 7	54 ± 7	0.005	0.311 to 1.555	0.937

They are reported as mean values ± standard deviation and compared between patients and controls. vmax, maximum velocity; LVEDVI, left ventricle end diastolic volume indexed; LVESVI, left ventricle end systolic volume indexed; LVEF, left ventricle ejection fraction; RVEDVI, right ventricle end diastolic volume indexed; RVESVI, right ventricle end systolic volume indexed; RVEF, right ventricle ejection fraction.

On CMR, *n* = 40 had PA regurgitation >10% and *n* = 31% > 25%. The right ventricles of the patients were dilated compared to controls, but the left ventricles in the rTOF group were within normal range for age (Table 1). In this age cohort, the normal values used at our institution limits normal CMR-derived LV end diastolic volume to 68–77 ml/m^2^ (girls 12–18 years) and 73–89 ml/m^2^ (boys 12–18 years) indicating no pathological enlargement of the LV in the rTOF group (18). With respect to the RV, the normal values for CMR-derived RV end diastolic volumes are 69–84 ml/m^2^ (girls 12–18 years) and 81–96 ml/m^2^ (boys 12–18 years), indicating a manifest dilatation of the RV in the rTOF group.

**Figure 2:**
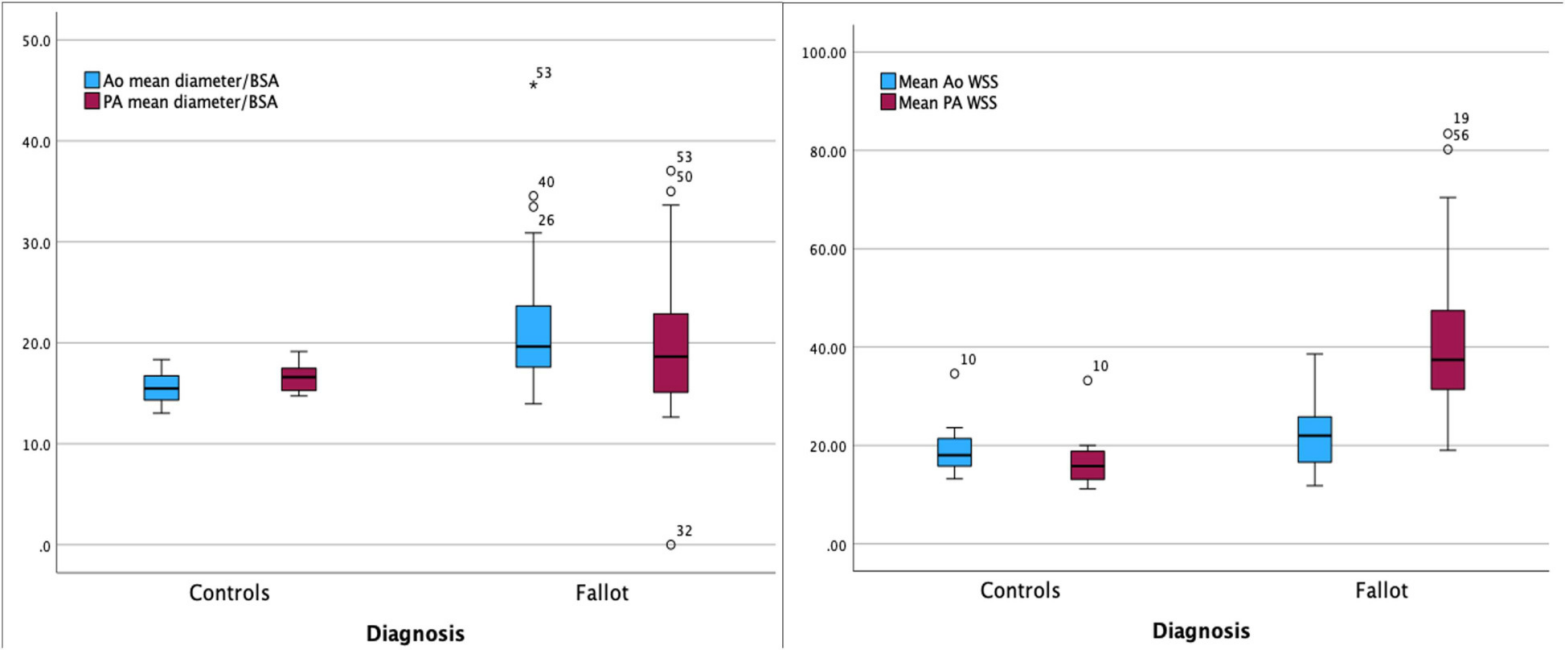
Boxplots comparing pulmonary artery and aortic diameter as well as wall shear stress in patients and controls. Left: the mean ascending aorta diameter per body surface area was significantly larger in the rTOF group than in controls (*p* < 0.001). rTOF patients also had a larger mean diameter of the pulmonary artery per body surface area than controls (*p* = 0.009). Right: The children with rTOF had higher levels of mean WSS in their pulmonary arteries, (*p* < 0.001) than controls while mean WSS in the ascending aorta was comparable to controls (*p* = 0.76).

Comparing peak and mean ascending aorta diameters/BSA between rTOF patients and controls revealed significantly larger vessel diameters in the rTOF group (both *p* < 0.001), as well as the mean PA diameter/BSA (*p* = 0.009) (Figure 2).

### Wall shear stress

Comparing the average of five measurements of peak and mean WSS in the PA, significant differences between rTOF patients and controls regarding both average peak WSS and average mean WSS, (average peak WSS 69 ± 13 cPa vs. 93 ± 29 cPa, *p* < 0.001 and average mean WSS 35 ± 13 cPa vs. 17 ± 5 cPa, *p* < 0.001) were revealed. There was also a wider distribution of WSS values in the rTOF group. Despite there being significant differences in aorta diameter/BSA, both peak and mean, between rTOF patients and controls, there was no significant difference in neither mean nor peak WSS in the ascending aorta (*p* = 0.14–0.76) (Figure 2).

When adjusting for age > or <10 years of age, no significant difference in WSS aorta or PA was revealed.

### Correlations

#### PA WSS vs. PA maximum flow velocity

Average WSS, both peak and mean, were strongly associated with the maximum velocity in the PA in rTOF patients (R = 0.84–0.91, *p* < 0.001), which is consistent with the way WSS is calculated from 4D flow CMR data, using velocity information in voxels near the wall in the vessel of interest.

#### PA diameter in rTOF patients vs. controls

**Figure 3:**
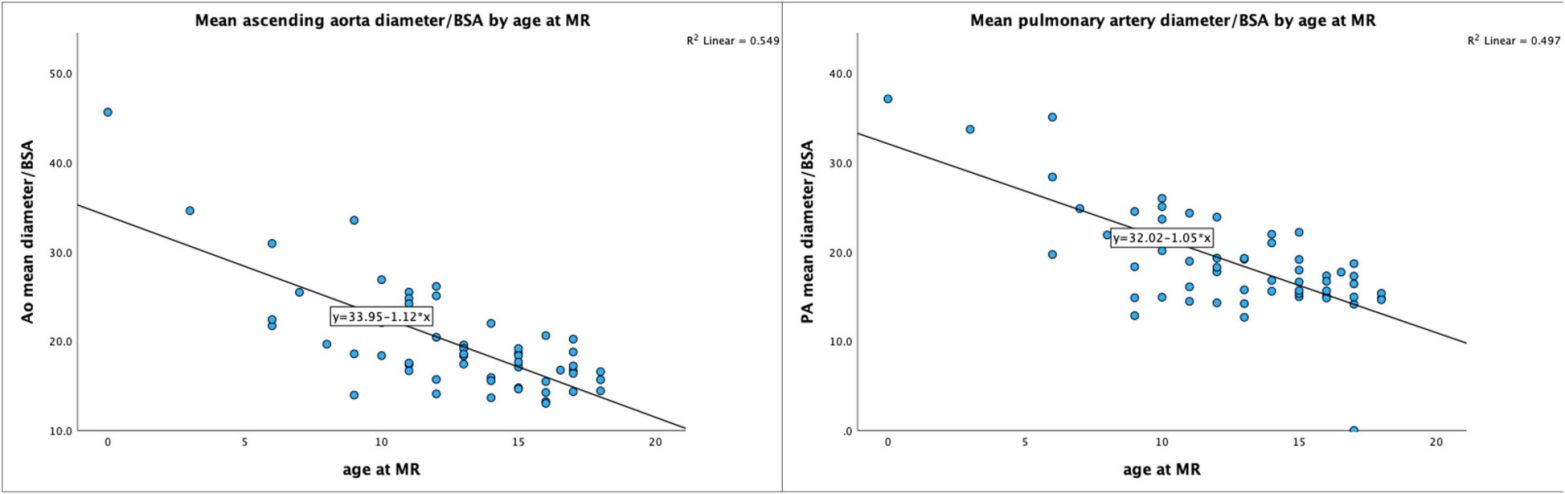
Scatterplots showing relationship between aortic and pulmonary artery diameters by age. In the rTOF group, there was a negative correlation between mean ascending aorta diameter/BSA and age (left), and likewise a negative correlation between mean pulomary artery diameter/BSA and age (right).

As shown in Table 1 and Figure 2, the mean PA diameter/BSA was significantly increased in the rTOF group compared to controls (*p* = 0.009), but not peak diameter/BSA (*p* = 0.09). The indexed PA diameter decreases with age in the rTOF group (R = −0.70 *p* < 0.001) (Figure 3), but no such correlation was seen in the control group (R = −0.40, *p* = 0.14). In a subgroup with patients older than 10 years, (*n* = 27 in rTOF group, and *n* = 15 among controls), no significant differences could be seen in indexed PA diameters, neither peak nor mean (*p* = 0.69–0.74).

#### Ascending aorta diameter in rTOF patients vs. controls

In the aorta the mean diameter was increased in the rTOF group compared to controls (*p* < 0.001) and there was a negative correlation between mean aorta diameter/BSA and age (R = −0.57, *p* < 0.001; Figure 3). In the controls, there was no correlation between mean aorta diameter/BSA and age (R = −0.27, *p* = 0.34). There persisted a significant differences in indexed aorta diameter in the subgroup of patients older than 10 years [mean 19.2 ± 3.4 mm/m^2^, peak 20.2 ± 3.8 mm/m^2^ (rTOF) vs. peak 16.5 ± 1.8 mm/m^2^ and mean 15.5 ± 1.6 mm/m^2^ (controls), for both *p* < 0.001] (Figure 2).

#### PA WSS vs. RV volumes and RV function

In the whole rTOF group, no correlation between RVEDVI and mean or peak PA WSS was seen (R = 0.04–0.14, *p* = 0.39–0.8) nor to RVEF (R = −0.02–0.12, *p* = 0.48–0.9). In a subgroup with more advanced disease and RVEDVI > 140 ml/m^2^ (*n* = 10), the same correlations were analysed, and neither in that group was there any correlation between mean PA WSS and RVEDVI (R = −0.35, *p* = 0.33) or RVEF (R = 0.37–0.58, *p* = 0.06–0.27).

#### PA WSS vs. pulmonary artery diameter and regurgitant fraction

There were no significant correlations between peak WSS or mean WSS and PA diameter (R = −0.27–0.06, *p* = 0.21–0.88) in the rTOF group. Neither were there any correlations between average peak WSS/average mean WSS and the PA regurgitant fraction (R = 0.31–0.35, *p* = 0.1–0.15) in this patient group. The subgroup analysis including only the rTOF patients with a RVEDVI > 140 ml/m^2^ (*n* = 10) and thus approaching the volumetric criteria for PVR, did not reveal significant correlations between peak PA WSS and peak PA diameter/BSA (R = −0.32, *p* = 0.34) or between PA WSS and PA regurgitant fraction (R = 0.24, *p* = 0.48).

## Discussion

In this retrospective review of patients with rTOF and controls, we systematically measured flow dynamics with CMR 4D flow derived WSS in the Ao and PA as well as vessel diameters and ventricular function. The patients in the rTOF group already during childhood had a manifest enlargement of their RV that according to present guidelines would warrant investigation for possible pulmonary valve replacement. The most important findings were that the patients in the rTOF group demonstrated high levels of WSS in the PA but, contrary to what we expected, there was no significant correlation to either PA dilatation, regurgitation or increased RV volume.

### 4D flow wall shear stress and vascular remodelling in rTOF

In recent years, WSS has been suggested to constitute a risk marker for vascular remodelling and later development of atherosclerotic disease and aneurysms in the systemic circulation (19, 20). The WSS is reported to regulate transcription in vascular remodelling (21), and high levels of WSS have predominantly been shown to aggravate manifest atherosclerosis (22). Which roles these pathways play in the pathogenesis of long-term complications in patients with CHD is still unknown.

WSS can be calculated in different ways; from echocardiography or from using computational fluid dynamics (CFD) from computed tomography or CMR (23, 24). CMR 4D flow yields information about the velocity gradients in blood adjacent to the vessel wall, and the shear force can be calculated by commercially available software. In many institutions, 4D flow is now a standard part of clinical CMR analysis for CHD. The litterature further highlights the potential of WSS, currently mostly applied in research, to become a clinically useful biomarker (25–27). However, determining WSS based on CMR 4D flow has disadvantages; there is a tendency towards underestimation because of limited spatial and temporal resolution (28). Also, in complex flows the range of velocities in each voxel can be wide and vary between individuals. In this study, where many of the patients were expected to display regurgitant flow, we used circumferential WSS which is more sensitive to helical or turbulent flow in the transverse direction of the vessel, rather than longitudinal WSS which is more sensitive to shear forces in the longitudinal axis, as would be expected in a stenosis.

### Wall shear stress and diameters in the ascending aorta and pulmonary artery

Concerning the aorta, WSS has been studied in mainly bicuspid aortic valve and Marfan syndrome (29, 30), where it has been shown to predict progressive dilatation (31). One CMR 4D flow study by Schäfer et al. (32) has focused on the proximal thoracic aorta of adolescents with rTOF repaired in infancy and shows many similarities to our study, concerning patient age and study group composition. We found significant dilatation of the ascending aorta in the rTOF patients, whereas Schäfer et al. did not. On the other hand, they found elevated levels of WSS in the ascending aorta, whereas we did not. Despite difference in findings, our conclusions were similar; that patients that have undergone early repair of TOF are at risk of late aortic complications. In addition we found a decreasing indexed ascending aorta diameter with age. While the study by François et al. relies exclusively on echocardiography data and therefore cannot be fully translated to our work, it similarly demonstrates a successive normalization of indexed aortic diameters in rTOF patients who underwent corrective surgery in infancy, effectively halting progressive dilatation of the ascending aorta (33). Other rTOF studies have shown normalized aortic diameters in mid childhood (34, 35), which is contrary to our results; even though median age for repair in our study was 5 months, there persisted a significant difference in indexed ascending aorta diameter in patients older than 10 years. This is an important finding, and although the study has a cross-sectional design, it suggest that early repair does not necessarily protect against ascending aortic dilatation. Although different studies have shown various results, aortic diameter may possibly need surveillance in this patient group. Several studies have focused on WSS in the PA, mainly in patients with pulmonary arterial hypertension (36, 37). Only a few studies have evaluated WSS after TOF repair; these have shown that patients with repaired TOF exhibit elevated levels of WSS in the PA and its branches (38, 39). Rizk et al. examined adult patients and included 24 patients with rTOF and 11 controls, where only 17 of the patients hade undergone the TAP repair (14). They found circumferential WSS (both peak and mean) significantly higher in the PA of patients with rTOF than in controls. Hudani (39) also studied adults, 17 patients and 20 controls, and in concordance with our study found significantly elevated levels of circumferential WSS along the PA compared to controls. One pediatric rTOF study by Hu et al. (40) where 25 patients and 10 controls were younger (8.44 ± 4.52 and 8.2 ± 1.22 respectively) than in our study, also revealed increased levels of PA WSS. The above studies have shown only weak or no correalations between PA regurgitant fraction an PA WSS, which is in line with our results in adolescent patients.

Our cross sectional study included all patients that were examined at our institution during the relevant time period without excluding those with stenoses, competent valves and importantly; those who had already undergone pulmonary valve replacement. This is both a strength and a weakness in our study; patients with rTOF display different pathologic traits and highly variable disease progressions. This highlights the difficulties in displaying how PA regurgitation influences WSS in a clinical setting.

Moreover, WSS is sensitive to differences in flow that are not described in simplistic terms of velocity or regurgitation. WSS is affected by spatial and temporal resolution (especially important in a pediatric cohort), respiratory motion and field strength (41). We have used both 1.5T and 3T in clinical practice with the majority (35/43) examined on 3T, which may have slightly influenced our results (42). Inter-observer variability and reproducibility also limit how WSS can be implemented in clinical routine. A combination of all the above mentioned probably partly explain our findings. In some cases though there were trends that did not reach significance, for instance RVEF vs. PA WSS and PA regurgitant fraction vs. PA WSS. Using small patient cohorts always pose a risk of type 2-errors where true correlations goes unnoticed. We are aware of this drawback with our study and hope to be able to contribute to prospective research in this area. There are still no longitudinal studies showing how elevated WSS in the PA of rTOF patients can predict vascular remodelling and future need for additional interventions.

While WSS has been shown to influence the risk for vascular remodelling in the systemic circulation; in the PA the relationship remains to be elucidated. In patients with rTOF, RVOT and PA are often wide due to earlier surgery, e.g., TAP, as in most of the patients (*n* = 35/43) in our study. Interestingly, there were no significant differences in PA diameter in the rTOF patients older than 10 years and controls, which could be attributed to a larger proportion of patients having had reinterventions in the older cohort. In our study, there was no correlation between degree of dilatation and levels of WSS in the PA. Our patients were on average 11.6 years old, and we know that in rTOF, vessel dilatation progresses over a long period of time. Even though other research groups have shown correlations between WSS and regurgitation, our patients may have been too early in their disease progression to exhibit sufficient degrees of dilatation or regurgitation. In support of this theory, the patients did not display left ventricular enlargement or reduced RVEF. Against that reasoning stands that the average rTOF patient in our study had a right ventricle enlargement, indicating that the rTOF patients were on average on an advanced stage in their disease progression.

Further studies are required to clarify the relationship between elevated WSS in the pulmonary artery and dilatation/regurgitation, as well as between WSS and ascending aortic dilatation in rTOF patients. Moreover, the lack of studies correlating WSS with clinical endpoints must be addressed before WSS can be integrated into clinical management guidelines.

### Limitations

Our sample was small, and the rTOF group was also somewhat heterogenous concerning the method and timing of repair, although the majority of patients had undergone the TAP at some point, that could introduce bias to the results. The control group included slightly older individuals which can also introduce an uncertainty regarding vascular remodelling that could potentially be more advanced in an older age group. However, these are slowly developing changes and the few years of difference in age is probably not significant (43).

## Conclusions

Pediatric patients with rToF revealed increased ventricular volumes, Ao/PA diameter and PA WSS compared to controls. Unexpectedly no association of WSS to Ao/PA dilatation, PA regurgitation or ventricular volumes, could be determined. Vascular remodelling in patients with rTOF is undoubtedly multifactorial, but increased WSS could be a contributing factor to late complications in rToF. WSS remains to be evaluated longitudinally and to be related to clinical outcome.

## Data Availability

The raw data supporting the conclusions of this article will be made available by the authors, upon request and without undue reservation.
