# Supplementary material for: Cardiovascular magnetic resonance 4D flow derived aortic and pulmonary wall shear stress in pediatric patients with repaired tetralogy of Fallot
Source: Front Pediatr. 2025 Oct 30;13:1623218. doi: 10.3389/fped.2025.1623218 (PMC12611806; doi:10.3389/fped.2025.1623218)
Supplement: Supplementary file 1 [file Supplementaryfile1.docx]

**Supplement 1**

The MRI protocol we have been using is as follows:

For ventricular function assessment, standard ECG-gated cine balanced steady-state free precession (bSSFP) sequences were used, with a 4-8 mm slice thickness in two-chamber, 4 chamber-, RVOT and short axis views. The 4D flow scan protocol included an ECG-gated, respiratory compensated and motion-compensated four-dimensional (4D) PC CMR flow protocol with TR/TE = 4.0-4.4 ms/2.2-2.3 ms, flip angle 15°, receiver bandwidth 31.25 kHz; field-of-view 360-440 x 360-440 mm², acquisition voxel size 2x2x2 – 2.2x2.2x2.2 mm³ and number of excitations 4, hypercat acceleration 6-8x and temporal resolution 31-63 ms. Velocity encoding (VENC) was optimized to avoid velocity aliasing, based on the maximum velocity in the relevant vessels measured with echocardiography, and chosen to be close to the maximum expected velocity. Contrast media, Gadoterate meglumine, Guerbet Princeton, NJ, USA, was administered intravenously at a dose of 0.02 mmol/kg and was used where clinically indicated, for anatomical evaluation with 3D whole heart b-SSFP and tissue characterization with late gadolinium enhancement (LGE) and/or T1 mapping. The 4D flow acquisition was performed immediately after the 3D whole heart.
